# Deciphering Molecular Mechanisms Governing the Reproductive Molt of *Macrobrachium nipponense*: A Transcriptome Analysis of Ovaries across Various Molting Stages

**DOI:** 10.3390/ijms241311056

**Published:** 2023-07-04

**Authors:** Huwei Yuan, Zijian Gao, Pengfei Cai, Wenyi Zhang, Shubo Jin, Sufei Jiang, Yiwei Xiong, Yongsheng Gong, Hui Qiao, Hongtuo Fu

**Affiliations:** 1 Wuxi Fisheries College, Nanjing Agricultural University, Wuxi 214081, China; yuan08102021@126.com (H.Y.);; 2 Key Laboratory of Freshwater Fisheries and Germplasm Resources Utilization, Ministry of Agriculture and Rural Affairs, Freshwater Fisheries Research Center, Chinese Academy of Fishery Sciences, Wuxi 214081, China

**Keywords:** transcriptome, molting, ovary, *Macrobrachium nipponense*

## Abstract

The relationship between molting and reproduction has received more attention in economically important crustacean decapods. Molting and reproduction are synergistic events in *Macrobrachium nipponense*, but the molecular regulatory mechanisms behind them are unclear. In the current study, we performed Illumina sequencing for the ovaries of *M. nipponense* during the molt cycle (pre-molting, Prm; mid-molting, Mm; and post-molting, Pom). A total of 66.57 Gb of transcriptome data were generated through sequencing, resulting in the identification of 105,149 unigenes whose alignment ratio with the reference genome exceeded 87.57%. Differentially expressed genes (DEGs) were annotated through the Gene Ontology (GO) and Kyoto Encyclopedia of Genes and Genomes (KEGG) databases for gene classification and pathway analysis. A total of twenty-six molt-related DEGs were found, and their expression patterns were examined across various molting stages. The KEGG enrichment analysis revealed that the key pathways involved in regulating the molting process of *M. nipponense* primarily include the mTOR, insect hormone biosynthesis, TGF-beta, and Wnt signaling pathways. Our transcriptomic data suggest that these pathways crosstalk with each other to regulate the synthesis and degradation of ecdysone throughout the molt cycle. The current study has deepened our understanding of the molecular mechanisms of crustacean molting and will serve as a basis for future studies of crustaceans and other molting animals.

## 1. Introduction

Successful molting is essential for the survival, growth, and reproduction of crustaceans. A complete molting procedure includes developing a fresh cuticle and shedding the old one, as well as strengthening and tanning the newly formed cuticle [1]. Crustacean molt is regulated by a complex endocrine system that secretes multiple hormones. Of these, 20-hydroxyecdysone (20E) is the key hormone to induce molting. In crustaceans, the Y-organ serves as the primary site for catalytic synthesis of ecdysteroids, making it a pivotal focus in research on the mechanisms underlying the molting process. However, studies have shown that molting is inextricably linked to reproduction in most crustaceans during the breeding period. The association between molting and reproduction is particularly evident in female crustaceans, primarily due to the cyclic development of their ovaries. However, the regulatory mechanisms governing the connection between molting and reproduction in crustaceans have not been fully elucidated.

The relationship between molting and reproduction exhibits variation across different crustacean species. In general, cirripedes undergo a relatively rapid molting process and their reproduction may necessitate multiple molting cycles beforehand to complete successfully. Crabs and lobsters experience a lengthier molting process, which overlaps with their reproductive period. Interestingly, isopods and shrimps exhibit a unique phenomenon where molting and reproduction occur simultaneously [2]. However, the relationship between reproduction and molting in crustaceans may vary. For example, *Paratelphusa hydrodromus* does not molt during the breeding season [2]. In American Lobster (*Homarus americanus*) molting and reproduction are separate physiological events, which alternate [3]. In *Macrobrachium rosenbergii,* the molting and reproductive cycles exhibit an overlapping pattern [4]. In turn, molting and reproduction are synergistic events in *Macrobrachium nipponense*, meaning that they work together in a coordinated manner. According to the available research, the ovary maturation in *M. nipponense* completes during the pre-molting stage. Following molting, males engage in mating with females, and shortly after ovulation, the ovaries enter the recession stage [5,6].

The integration of high-throughput sequencing technology and gene annotation tools enables precise detection of transcript expression at different molting stages, serving as a powerful tool for studying dynamics of crustaceans’ molting processes. Presently, transcriptome analysis techniques are extensively employed in the investigation of crustacean molting. These studies have unveiled molting-related genes, hormone regulation genes, skelemin genes, immune-related genes, and significant pathways associated with molt regulation in crustaceans across various molting stages [7,8,9,10,11]. The diverse functions of the mechanistic target of the rapamycin (mTOR) signaling pathway in mammals have been thoroughly documented [12,13]. It is hypothesized that the mTOR pathway may play significant roles in crustacean molting. In *Gecarcinus lateralis*, the mTOR signaling pathway plays an important role in the activation of the Y-organ [11]. Specifically, *mTORC1* plays a crucial regulatory role in the production of ecdysteroids in crustacean Y-organs [14]. In insects and crustaceans, the transforming growth factor-beta (TGFβ) signaling pathway determines the responsiveness of the molting glands to neuropeptides [14,15]. In *Drosophila*, TGFβ is essential for ecdysone synthesis and developmental transition [16]. The TGFβ signaling is involved in the transition of the Y-organ from the activated to the committed state [17]. Ecdysone is one of the hormones synthesized by the insect hormone biosynthesis pathway, which is essential for molting [18]. The Halloween family genes catalyzing cholesterol synthesis of ecdysone directly affect arthropod molting [18]. Silencing the Halloween family genes leads to the failure of physiological functions such as molting and metamorphosis, and even death [6,18,19]. In addition, the main pathways involved in molting regulation, such as the Wnt signaling pathway, insulin signaling pathway, and calcium signaling pathway, among others, have been identified [20,21].

The relationship between molting and reproduction has received more attention in the commercially important crustacean decapods [2]. *M. nipponense* (Crustacea, Decapoda) is a widely recognized and economically significant prawn species in China [22]. As a popular food source, this species is consumed in China and is often considered a delicacy due to its unique flavor and texture. Furthermore, the species’ high market demand and fast growth rate make it attractive for aquaculture production. Cultivation of this species can provide a source of income for farmers and help to meet the growing demand for aquatic products in many parts of the world. The present study specifically investigated the association between ovary maturation and molting processes in *M. nipponense*. For this purpose, the ovaries’ transcriptome library of the *M. nipponense* in three different molting stages (pre-molting: Prm, mid-molting: Mm, and post-molting: Pom) was constructed using the Illumina sequencing platform. The current study focuses on screening for molting-related genes and pathways, thereby improving our understanding of the molecular mechanisms responsible for reproductive molting in *M. nipponense*.

## 2. Results

### 2.1. Sequencing Data and Comparative Efficiency

A total of 66.57 Gb of transcriptomic data were obtained from nine ovarian samples at different molting periods (pre-molting, mid-molting, and post-molting) by sequencing, with clean reads of >6.5 Gb for each sample. A total of 221,894,778 pin-end reads, 161,531 transcripts and 105,149 unigenes were obtained. The Q30 values of the groups ranged from 91.15% to 92.77%, and the comparison rate with the reference genome reached more than 87.57% (Table 1). All sequence reads are available in the National Center for Biotechnology Information (NCBI) Sequence Read Archive (accession number: PRJNA977108).

### 2.2. Identification and Annotation of Differentially Expressed Genes (DEGs)

**Figure 1:**
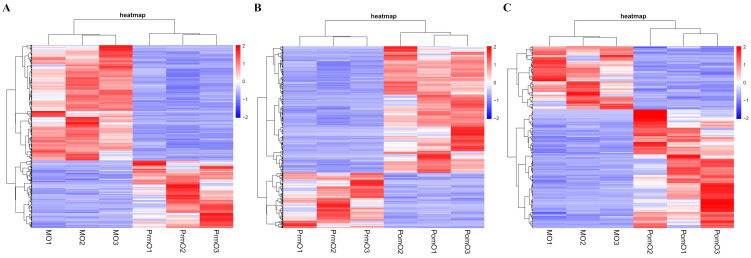
Clustering diagram of DEGs. The horizontal coordinates represent the sample names and the clustering results of the samples, and the vertical coordinates represent the DEGs and the clustering results of the DEGs. The colors represent the expression levels of DEGs in the samples. PrmO VS MO (**A**); PrmO VS PomO (**B**); MO VS PomO (**C**).

Genes with a fold-change ≥ 2 and an FDR < 0.05 were identified as DEGs by comparing different molt stages. The number of DEGs for PrmO vs. MO was 1663, including 1049 up-regulated and 614 down-regulated DEGs; PrmO vs. PomO has 2655 DEGs, 1845 up-regulated and 810 down-regulated DEGs; MO vs. PomO has 348 DEGs, 227 up-regulated and 121 down-regulated DEGs (Figure 1).

The DEGs were functionally annotated in different databases (Swiss-Prot, GO, KEGG, COG, KOG, Pfam and NR) and a total of 3893 DEGs were annotated in seven databases. Of these, a total of 2970 DEGs were annotated in the Swiss-Prot database, 2773 DEGs were annotated in the GO database, 1914 DEGs were annotated in the KEGG database, 902 DEGs were annotated in the COG database, 2341 DEGs were annotated in the KOG database, 2723 DEGs were annotated in the Pfam database, and 2301 DEGs were annotated in the NR database (Table 2).

### 2.3. GO Enrichment and Pathway Annotation of DEGs

**Figure 2:**
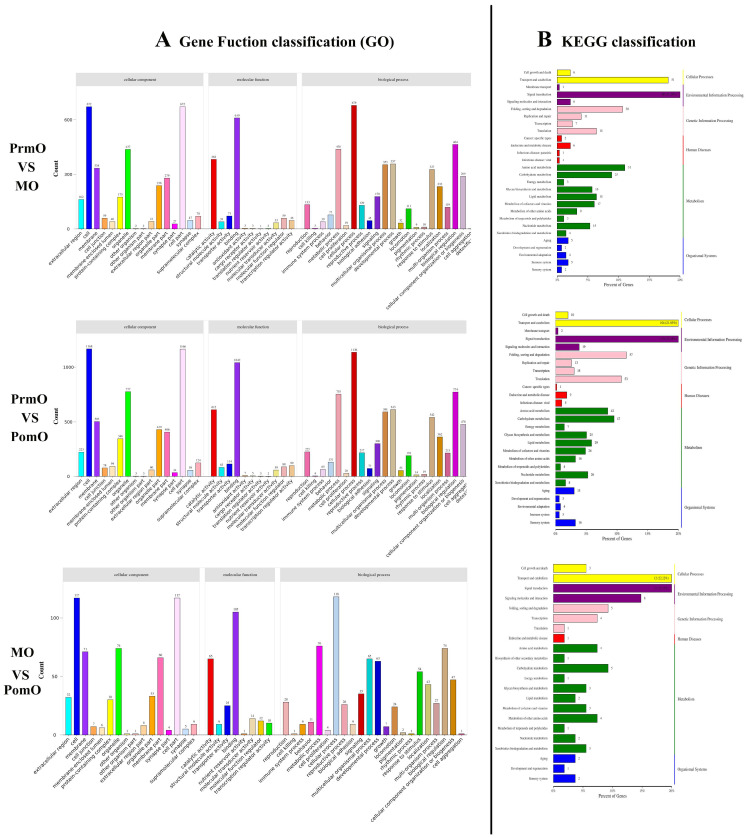
GO and KEGG classification of all DEGs. (**A**) The x-axis is the GO classification and the y-axis is the number of genes, (**B**) the y-axis (left) is the name of KEGG secondary metabolic pathway, the y-axis (right) is the name of KEGG primary metabolic pathway, and the x-axis is the number of genes annotated to this pathway and their proportion to the total number of annotated genes.

DEGs were annotated through GO and KEGG databases for gene classification and pathway analysis. A total of 2773 transcripts were assigned to at least one of the three GO terms by GO database annotation (Figure 2A). Within the cellular component category, the representative GO terms are “cell” and “cell part”. In the category of molecular function, the representative GO terms are “binding” and “catalytic activity”. “Cellular processes”, “biological regulation”, and “metabolic processes” are well-represented terms in the category of biological process.

The annotation analysis of the DEGs’ pathways is helpful to further understand the function of genes. KEGG analysis showed that a total of 1914 DEGs were annotated to 309 signaling pathways (Figure 2B). Among these annotated pathways, those classified as related to “transport and catabolism” and “signal transduction” account for more than 40%. In addition, the dominant pathways include “folding, sorting and degradation”, “translation”, “amino acid metabolism”, and “carbohydrate metabolism”.

### 2.4. qRT-PCR Validation of DEGs

**Figure 3:**
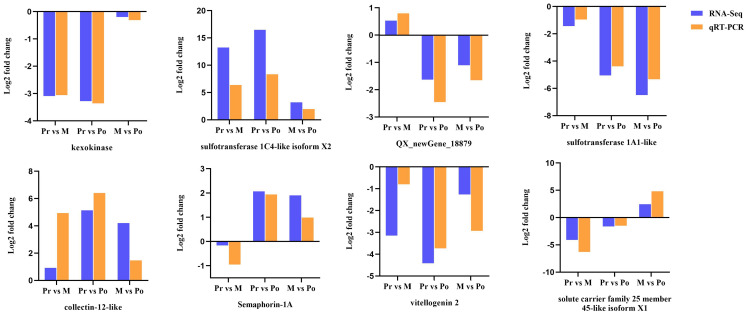
Expression patterns of 8 DEGs from RNA-seq (blue) and qRT-PCR (orange) in different molting stages.

To verify the reliability of RNA-Seq data, eight DEGs were randomly selected, and their expression levels were detected by qRT-PCR at different molting stages. The comparative results among different groups show that the genes *kexokinase*, *sulfotransferase 1A1-like*, and *vitellogenin 2* were downregulated, while the genes *sulfotransferase 1C4-like isoform X2* and *collectin-12-like* were upregulated. The results showed that the expression patterns of these eight DEGs detected by qRT-PCR were consistent with the results of RNA-Seq data, indicating that the results of our transcriptome data were reliable (Figure 3).

### 2.5. Candidate DEGs Associated with Molting and Their Expression Pattern

**Figure 4:**
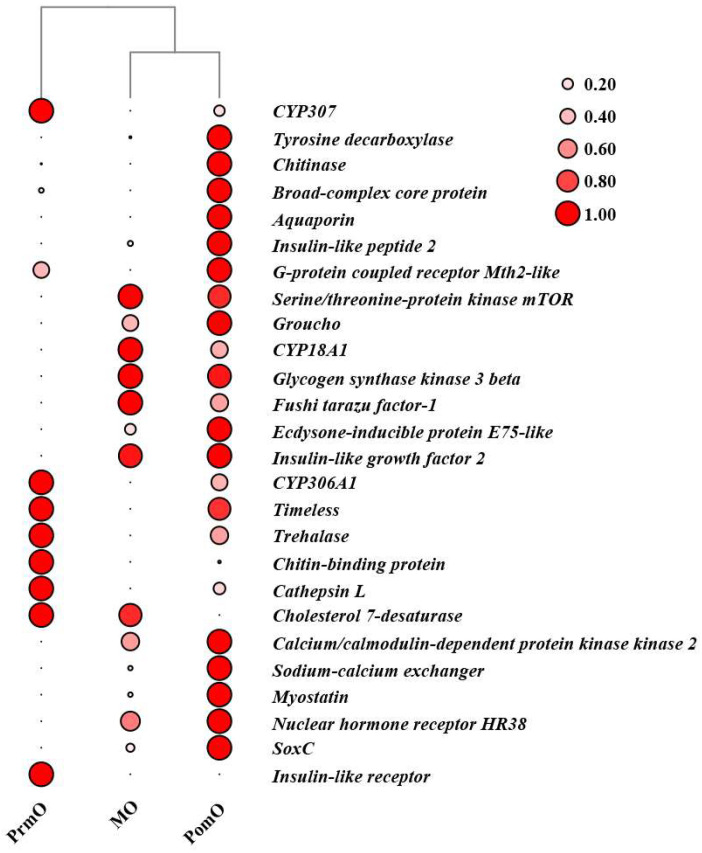
Heatmap showing the abundance of the expression of genes associated with molting. The abundance of gene expression was expressed as FPKM (fragments per kilobase million). Clustering of the genes during the three different molting stages.

By comparing and analyzing ovarian transcriptome at different molting stages, 26 molting-related genes were obtained. Eight genes (*CYP307*, *CYP306A1*, *Timeless*, *Trehalase*, *Chitin-binding protein*, *Cathepsin L*, *Cholesterol 7-desaturase*, *Insulin-like receptor*) were highly expressed at the pre-molting stage, with similar expression patterns. Five genes (*Serine/threonine-protein kinase mTOR*, *CYP18A1*, *glycogen synthase kinase 3 beta*, *Fushi tarazu factor-1*, *Insulin-like growth factor 2*) were highly expressed in the mid-molting stage. Thirteen genes (*Tyrosine decarboxylase*, *Chitinase*, *Broad-complex core protein*, *Aquaporin*, *Insulin-like peptide 2*, *G-protein coupled receptor Mth2-like*, *Groucho*, *Ecdysone-inducible protein E75-like*, *Calcium/calmodulin-dependent protein kinase kinase 2*, *Sodium-calcium exchanger*, *Myostatin*, *Nuclear hormone receptor HR38*, *SoxC*) were highly expressed at the post-molting stage. The expression patterns and clustering of these genes were revealed through their heat map (Figure 4).

### 2.6. Analysis of DEGs in Signaling Pathways Involved in Molting Regulation

**Figure 5:**
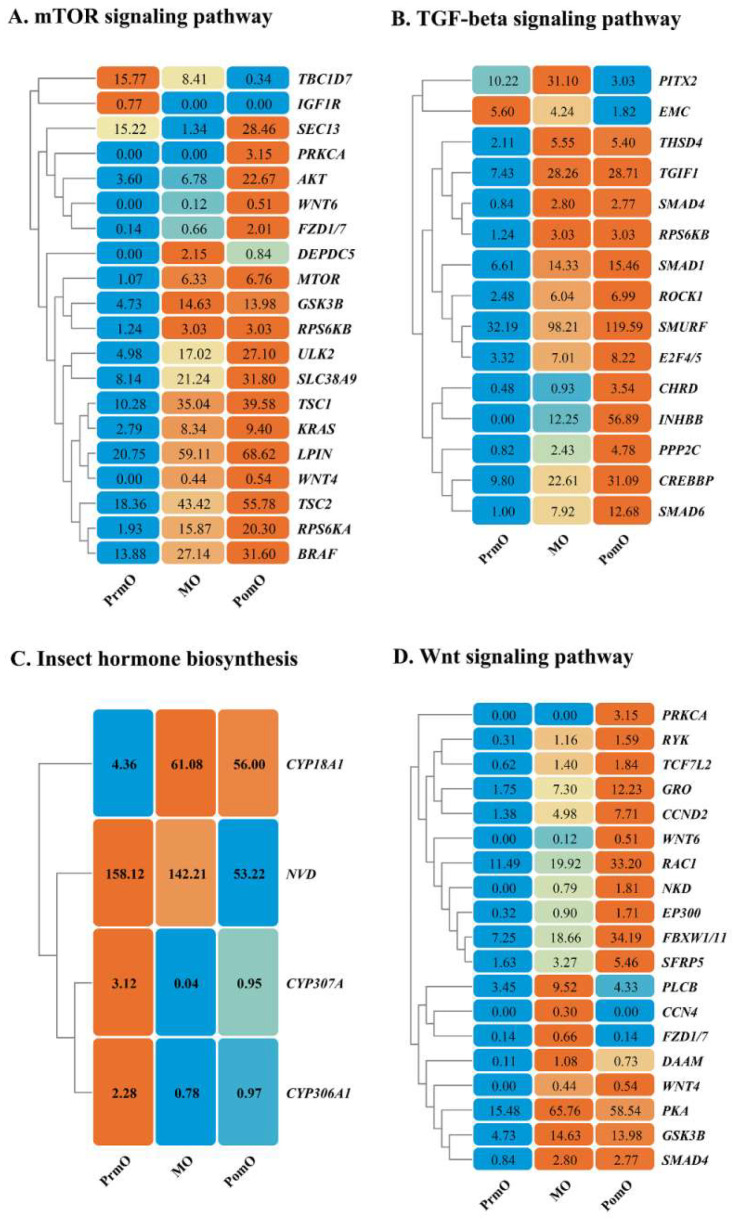
Heatmaps illustrating the expression abundance of DEGs within signaling pathways implicated in the regulation of molting process. (**A**) The heatmap of mTOR signaling pathway. (**B**) The heatmap of TGF-beta signaling pathway. (**C**) The heatmap of insect hormone biosynthesis. (**D**) The heatmap of Wnt signaling pathway. The values in the figure indicate FPKM.

In order to investigate the role of signaling pathways involved in the regulation of the molting process, a clustering analysis on the expression patterns of relevant DEGs was performed. We identified 20 DEGs in the mTOR signaling pathway. The two genes (*TBC1D7* and *IGF1R*) showed the same expression pattern and were highly expressed at the pre-molting stage. Three genes (*DEPDC5*, *GSK3B,* and *RPS6KB*) were highly expressed at the mid-molting stage. In addition, a total of fifteen genes (*SEC13*, *RPS6KA*, *MTOR*, *TSC1*, *TSC2*, *KRAS*, *ULK2*, *LPIN*, *WNT6*, *PRKCA*, *WNT4*, *BRAF*, *SLC38A9*, *FZD1/7*, *AKT*) had the highest expression levels at the post-molting stage (Figure 5A).

Fifteen DEGs were identified in the TGF-beta signaling pathway. *PITX2* and *EMC* genes had the lowest expression levels at the post-molting stage. The expression levels of other DEGs (*CHRD*, *INHBB*, *CREBBP*, *SMAD4*, *SMAD6*, *SMURF*, *RPS6KB*, *TGIF1*, *THSD4*, *PPP2C*, *ROCK1*, *SMAD1*, *E2F4/5*) in the pathway were lowest at the pre-molting stage (Figure 5B).

Four DEGs were identified in insect hormone biosynthesis, among which *CYP307A* and *CYP306A1* had the same expression pattern, and the expression level was the highest at the pre-molting stage. *NVD* gene was highly expressed at the pre-molting stage and the mid-molting stage, while *CYP18A1* gene was highly expressed at the mid-molting stage and the post-molting stage (Figure 5C).

A total of 19 DEGs were enriched in the Wnt signaling pathway. Among them, 12 DEGs (*PRKCA*, *RYK*, *TCF7L2*, *GRO*, *CCND2*, *WNT6*, *RAC1*, *NKD*, *EP300*, *FBXW1/11*, *SFRP5*, *WNT4*) showed the same expression pattern, which gradually increased from the pre-molting to the post-molting stage. The other seven DEGs (*PLCB*, *CCN4*, *FZD1/7*, *DAAM*, *PKA*, *GSK3B*, *SMAD4*) showed the same expression pattern, increasing from the pre-molting to the mid-molting stage and decreasing at the post-molting stage (Figure 5D).

### 2.7. Expression of Various Molting-Related Genes at Different Molting Stages

**Figure 6:**
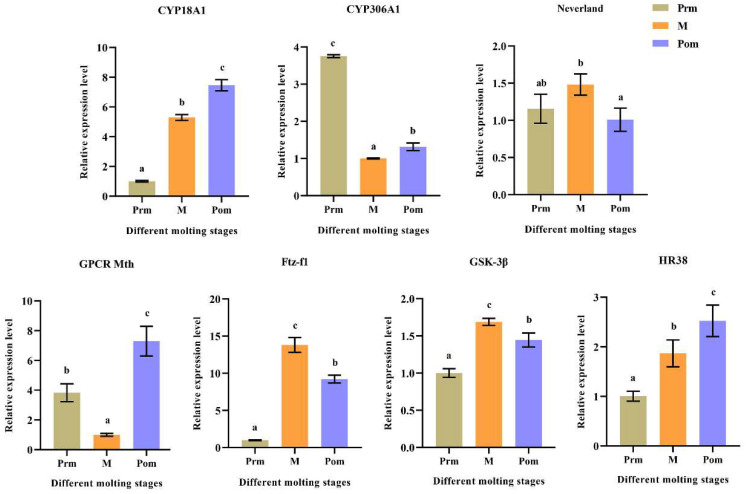
The qRT-PCR results of molting-related genes at different molting stages. Prm, pre-molting; M, mid-molting; Pom, post-molting. Data are expressed as mean ± SD (n = 6). Bars with different letters indicate significant differences (*p* < 0.05).

The expression levels of seven strong candidate DEGs related to molting were examined by qRT-PCR at different molting stages of *M. nipponense* (Figure 6). The expression levels of the three genes (*CYP18A1*, *GPCR Mth*, *HR38*) were the highest at the post-molting stage, among which *CYP18A1* and *HR38* had the same expression pattern, and their expression levels gradually increased from the pre-molting to post-molting stages. The expression level of *CYP306A1* was the highest at the pre-molting stage and the lowest at the mid-molting stage. The expression level of *Neverland* in the mid-molting stage was significantly higher than that in the post-molting stage. The expression levels of *Ftz-f1* and *GSK-3β* increased from the pre-molting stage to the mid-molting stage and then decreased.

## 3. Discussion

Molting is a critical process for crustaceans, and the cyclic nature of this process has been the subject of extensive research. In this study, the ovarian transcriptome of *M. nipponense* was assembled at different molting stages to screen out the important molting-related pathways and DEGs. Comparing different molting stages, we observed a greater abundance of DEGs during the transition from Prm to Mm and Pom, indicating their pivotal role in the molting process. The subsequent analysis focused on the function characteristics of those molting-related genes in *M. nipponense* at various molting stages.

In crustaceans, *Neverland* plays a crucial role in molting by regulating the synthesis of ecdysteroids [23,24]. Transcriptome results, as recorded in our study, indicate that *Neverland* is highly expressed in both the Prm and Mm stages, and may play an important role in both stages. The Halloween family gene *CYP306A1* plays an indispensable role in the synthesis of 20E by catalyzing the conversion of ketodiol into ketotriol [6]. In *M. nipponense*, *CYP306A1* knockdown significantly reduced 20E content and molting frequency [25]. In the current study, *CYP306A1* was highly expressed at the pre-molting stage, suggesting that *CYP306A1* may play a role in 20E synthesis, which is consistent with previous studies [25]. In insects, *CYP18A1* encodes a cytochrome P450 enzyme with 26-hydroxylase activity that inactivates steroid hormones [26]. The nuclear receptor genes *Ftz-f1* and *HR38* encode cuticular proteins, which are directly regulated by ecdysone [27]. *Ftz-f1* plays a pivotal role in the molting and ovulation process of *M. nipponense* [28]. In *Drosophila* metagenesis, the formation of the cuticle requires the function of *HR38*, which the mutation of leads to the rupturing of the cuticle [29]. Glycogen synthase kinase 3β (*GSK-3β*) functions in many cellular processes, including the regulation of different cellular signals, cell differentiation, growth, and apoptosis [30,31]. In *Litopenaeus vannamei*, knocking down the *GSK-3* gene can affect the growth rate of shrimp, suggesting that it plays a vital role in molting regulation [32]. G-protein coupled receptors (GPCRs) are an ancient and ubiquitous family of proteins. During the process of insect molting, the expression levels of GPCR-1 are increased, primarily under the regulation of 20E. Moreover, GPCR-1 plays a critical role in the transformation from the larval to pupal stages [33]. In crustaceans, GPCR genes are involved in molting, vitellogenesis and other processes [34,35]. In *G. lateralis*, three GPCRs are differentially expressed at different molting periods, suggesting that they are associated with the regulation of ecdysteroid production [34]. In the present study, there were significant differences in the expression levels of G-protein coupled receptor Methuselah (*GPCR Mth*) at different ecdysis stages of *M. nipponense*, suggesting that *GPCR Mth* may play an important role in molting regulation.

Performed during the current study, transcriptome analysis identified several signaling pathways involved in the regulation of crustacean molting, including the mTOR, TGF-beta, insect hormone biosynthesis, and Wnt signaling pathways. The mTOR signaling pathway plays an important role in the balance between survival and reproduction, and is required for uninterrupted ecdysone synthesis [10,36]. Many DEGs in the pathway are associated with molting and growth, such as the *mTORC1* gene which is necessary for arthropod ecdysone production [37]. In *Drosophila*, the *TSC2* gene is associated with larval development, and knocking down the gene leads to lower body weight [38]. Consistent with our results, the expression levels of *MTOR* and *AKT* genes were increased during the mid- and post-molting stages of *G. lateralis* [39]. The TGF-beta signaling pathway also performs an important function during molting and is required for the sustained upregulation of mTOR signaling during the pre-molting period [40]. TGF-beta signaling is associated with uninterrupted production of ecdysteroids, mediating the transition of YO from an activated to a committed state [39]. In *G. lateralis*, inhibition of TGF-beta signaling leads to reduced production of ecdysteroids [41]. The expression pattern of common DEGs screened in the enriched TGF-beta signaling pathway in the previous study was identical to our results [11]. Insect hormone biosynthesis regulates the synthesis of sesquiterpenoid juvenile hormones (JH) and steroidal ecdysteroids that are essential for arthropod development and reproduction [42]. Our transcriptome analysis reveals that DEGs such as *NVD*, *CYP307A*, *CYP306A1*, and *CYP18A1* within the insect hormone biosynthesis pathway play critical roles in the synthesis or inactivation of steroidal ecdysteroids. Firstly, the enzyme encoded by *NVD* catalyzes the conversion of cholesterol to 7-dehydrocholesterol, then 7-dehydrocholesterol is converted to 20E catalyzed by the Halloween family gene, and finally, the enzyme encoded by *CYP18A1* catalyzes the oxidation of 20E to 26-carboxylic acids [1,6]. The Wnt signaling pathway is implicated in a diverse array of biological processes, encompassing cell proliferation and differentiation, embryonic morphogenesis, tissue regeneration, and the modulation of ecdysone biosynthesis [43,44]. In *G. lateralis*, many components of the enriched Wnt signaling pathway are differentially expressed during the molt cycle [10]. Previous studies have identified a large number of Wnt genes in crustaceans. These genes show different expression patterns in different molting stages of crustaceans and may play a role in molting and immune responses [45]. In insects, Wnt acts as an activator of *mTORC1* and indirectly regulates the production of ecdysone [46]. Taken together, our transcriptomic data suggest that various pathways crosstalk with each other to regulate the production of ecdysone and coordinate the growth and development of the organism.

In summary, the ovarian transcriptome of *M. nipponense* at different molting stages was assembled for the first time in this study. The current study reveals the molecular regulatory role of the ovaries in the molting cycle of *M. nipponense*. The identification of molting-related genes and pathways provides fundamental insights into the molecular mechanisms of molting in *M. nipponense* or other crustaceans and shed more light on the association between ovary maturation and the molting processes in crustaceans. The strong molt-related candidate DEGs identified by the transcriptome results will be the focus of our subsequent studies to reveal their role in the molting process. Furthermore, transcriptomic data demonstrate that multiple pathways are involved in regulating the synthesis and degradation of ecdysone throughout the molt cycle. One potential practical significance of these results is the development of more effective methods for controlling molting in crustacean aquaculture, which can improve growth rates and reduce mortality. Furthermore, understanding the molecular mechanisms underlying molting can provide insights into other physiological processes in crustaceans and other arthropods. Overall, this study provides important new insights into the molecular regulation of the molt cycle in crustaceans and has the potential to lead to practical applications in aquaculture and beyond.

## 4. Materials and Methods

### 4.1. Sample Collection and RNA Isolation

Healthy adult female prawns (2.10 ± 0.45 g) were collected from the Dapu experimental base, Wuxi, China, as described previously [8]. After one week of acclimatization under water recirculation conditions (26 ± 1 °C), ovarian tissues were collected from prawns at different molting stages. The molting stadium of the prawns was determined according to the criteria described in the previous study [47]. Ten prawn ovaries at pre-molting, mid-molting, post-molting stages were collected in triplicate, snap frozen by liquid nitrogen at −196 °C and stored at −80 °C for RNA extraction. Total RNA from prawn ovaries was isolated using the RNAiso Plus kit (TaKaRa, Shiga, Japan), according to the manufacturer’s instructions. RNA degradation and contamination were evaluated by 1.2% agarose gel electrophoresis. The purity and integrity of RNA were detected by Nanodrop ND2000 (NanoDrop Technologies, Wilmington, DE, USA) and Agilent 2100 (Agilent Technologies, Santa Clara, CA, USA), respectively.

### 4.2. The cDNA Library Construction and Sequencing

The construction and sequencing of cDNA libraries has been described in our recent study [8]. In brief, the method involved enriching the ovarian mRNA (3 μg) using magnetic beads coated with Oligo (dT) (Life Technologies, Carlsbad, CA, USA), followed by fragmentation of the mRNA. The first cDNA strand was synthesized with random hexamers using mRNA as a template, and then the second cDNA strand was synthesized by adding buffer, dNTPs, RNase H and DNA polymerase I. The cDNA was purified using AMPure XP beads (Beckman Coulter, Beverly, MA, USA). The cDNA underwent end-repair and poly (A) tails were added. Then, fragment size selection was performed with AMPure XP beads. Finally, the cDNA library was enriched by PCR and sequenced using Illumina HiSeq2500 (Illumina, San Diego, CA, USA).

### 4.3. Assembly and Gene Annotation

The raw data obtained by Illumina HiSeq 2500 high-throughput sequencing were filtered to obtain high-quality clean data. Mapped data was obtained by sequence alignment of clean data with the reference genome of *M. nipponense* (https://ftp.cngb.org/pub/CNSA/data2/CNP0001186/CNS0254395/CNA0014632/ (accessed on 9 September 2021)) by HISAT2 [48]. Transcripts were assembled from scratch using StringTie (http://ccb.jhu.edu/software/stringtie (accessed on 9 September 2021)) [49]. The expression level of transcripts or genes was measured by FPKM (fragments per kilobase of transcript per million fragments mapped) [50]. Differential expression analysis was performed using DESeq2 software (http://www.bioconductor.org/packages/release/bioc/html/DESeq2.html (accessed on 9 September 2021)) [51]. Genes with a fold-change ≥ 2 and false discovery rate (FDR) < 0.05 were regarded as differentially expressed. The differentially expressed genes (DEGs) were annotated in Nr (http://ncbi.nlm.nih.gov/ (accessed on 9 September 2021)), Gene Ontology (GO) (http://www.geneontology.org/ (accessed on 9 September 2021)), Kyoto Encyclopedia of Genes and Genomes (KEGG) (http://www.genome.jp/kegg/ (accessed on 9 September 2021)), and Clusters of Orthologous Group (COG) (http://www.ncbi.nlm.nih.gov/COG/ (accessed on 9 September 2021)) databases, respectively.

### 4.4. Quantitative Real-Time PCR (qRT-PCR) Validation

Eight randomly selected DEGs were examined using qRT-PCR to verify the accuracy of RNA-seq results. The reaction system and procedures for qRT-PCR are described in detail in our previous study [6]. All specific primers for qRT-PCR reactions were designed using primer5.0 and are listed in Table S1. The expression levels of examined genes were determined by the 2^−ΔΔCT^ method, with EIF as the internal reference gene [52,53]. The Tukey method was employed for one-way analysis of variance to compare differences among multiple groups. The results are presented as mean ± standard deviation (SD), and bars with different letters indicate significant differences (*p* < 0.05). Statistical analyses were performed using SPSS 20.0 software (IBM, New York, NY, USA).

## Figures and Tables

**Table 1:** Sequencing data statistics and quality control.

Sample	ReadSum	BaseSum	GC (%)	Q30 (%)	Reads Aligned (%)
PrmO1	22,839,573	6,851,871,900	39.49	92.23	87.57%
PrmO2	21,278,239	6,383,471,700	39.01	92.33	88.12%
PrmO3	21,613,754	6,484,126,200	39.11	92.77	88.35%
MO1	25,537,213	7,661,163,900	39.36	92.31	88.86%
MO2	20,883,459	6,265,037,700	40.07	91.15	88.30%
MO3	22,137,657	6,641,297,100	39.61	91.49	88.35%
PomO1	26,777,746	8,033,323,800	39.58	92.04	88.63%
PomO2	30,473,592	9,142,077,600	39.33	92.17	89.15%
PomO3	30,353,545	9,106,063,500	39.63	92.57	88.01%

**Table 2:** Differential gene annotation.

DEG_Set	Total	Swiss-Prot	GO	KEGG	COG	KOG	Pfam	NR
PrmO vs. MO	1388	1046	975	662	319	826	977	822
PrmO vs. PomO	2236	1720	1609	1139	521	1358	1558	1315
MO vs. PomO	269	204	189	113	62	157	188	164
Total	3893	2970	2773	1914	902	2341	2723	2301

## Data Availability

The authors confirm that the data supporting the findings of this study are available within the manuscript, Tables and Figures.

## Supplementary Materials

The following supporting information can be downloaded at: https://www.mdpi.com/article/10.3390/ijms241311056/s1.
